# The E23K Polymorphism of *KCNJ11* and Diabetic Retinopathy in Northern Iran

**DOI:** 10.3389/bjbs.2021.10245

**Published:** 2022-04-22

**Authors:** L. Alidoust, F. Ajamian, S. Abbaspour, A. Sharafshah, P. Keshavarz

**Affiliations:** ^1^ Department of Medical Genetics, Faculty of Medicine, Guilan University of Medical Sciences, Rasht, Iran; ^2^ Department of Biology, Faculty of Science, University of Guilan, Rasht, Iran; ^3^ Faculty of Medicine, Guilan University of Medical Sciences, Rasht, Iran; ^4^ Cellular and Molecular Research Center, School of Medicine, Guilan University of Medical Sciences, Rasht, Iran; ^5^ Division of Genetics, Department of Cell and Molecular Biology and Microbiology, Faculty of Science and Biotechnology, University of Isfahan, Isfahan, Iran

**Keywords:** KCNJ11, polymorphism, diabetic retinopathy, Iranian population, association study, c.67A>G

## Abstract

**Background:** Diabetic Retinopathy (DR) is one of the most severe micro-vascular complications of diabetes mellitus (DM), involving interactions between environmental and genetic risk factors. KCNJ11 gene has a key role in insulin secretion and is of substantial interest in various populations.

**Methods:** A population-based association of 524 T2DM patients was performed to delineate the genetic influence of KCNJ11 polymorphisms (rs5219, c.67A>G or E23K) on the risk of DR in an Iranian population. Genotyping was performed using TaqMan assay. Univariate and MLR analysis controlling for confounders was conducted to evaluate the association between rs5219 and DR.

**Results:** No significant difference was observed in either genotypes distribution (*p* = 0.83) or allele frequency (*p* = 0.66) between T2DM individuals with and without DR in any models of inheritance. Genotype-phenotype association showed that DR group carrying GA genotypes, a significantly higher mean age was observed compared with two other genotypes (*p* = 0.04). MLR analysis indicated that HbAlc with adjusted OR of 1.84 (95% CI, 1.46–2.33, *p* = 0.00) and first-degree relatives of family history with adjusted OR of 2.85 (95% CI, 1.45–5.58, *p* = 0.002) were significantly associated with DR, but the c.67A>G genotype is not an independent predictor of retinopathy.

**Conclusion:** Collectively, rs5219 was not associated with DR among Iranians with T2DM.

## Introduction

If unaddressed, diabetic retinopathy (DR) ultimately develops in about 95% of patients with type 1 diabetes mellitus (DM) and 60% of Type 2 Diabetes (T2D) patients, and it is the main cause of visual impairment (blindness) in adult people. Various studies of twins and families indicate an important role of genetic factors in association with DR (1, 2). Candidate genes analysis and Genome-wide association studies (GWAS) though meta-analysis have reported some potential susceptibility genes of DR (3). E23K (c.67A>G) is identified as a *KCNJ11* SNP associated with T2DM susceptibility in several populations, although with controversial results (4). Limited studies have been done on the relationship between T2DM susceptibility genes and DR that most of them included a relatively small sample size and only one locus or a few loci. In addition, gene analysis and GWAS in Chinese, Mexican-American and Caucasian populations indicated that several SNPs or loci have borderline or weak associations with DR in either type 1 or type 2 diabetes mellitus (3, 5, 6). Liu et al reported that rs5219 was associated with DR in the Chinese population with T2DM (7), but any role in a Caucasian population is unknown. Accordingly, we hypothesized that the E23K c.67A>G is linked to DR in a Caucasian population.

The hypothesis was tested in 524 unrelated T2DM patients, 234 without DR, 290 with. Informed consent was obtained from all participants. DR, of lack thereof, was diagnosed by standard retinal vessel analysis, and standard clinical, demographic and laboratory data were collected. This study was approved by the ethical committee for human genome/gene research at the Guilan University of Medical Science (IR.GUMS.REC.1394.341). Exclusion criteria were age <20 years, history of hematological diseases, hepatic disorders and malignancy, secondary diabetes such as chronic pancreatitis, Cushing’s disease, polycystic ovary disease, and drug induced diabetes. Genomic DNA was isolated from peripheral blood according to the standard salting-out method. Genotyping was performed by TaqMan assay technology on an ABI7300 system (Applied Biosystems, Foster City, CA). PCR program was set up at 95°C for 10 min, followed by 40 cycles of denaturation at 94°C for 15 s, annealing at 61°C for 1 min. All statistical analyses were performed using either the SPSS version 22.0 (SPSS Inc., Chicago, IL), the SNPAlyze software version 8.0 (Dynacom, Tokyo, Japan). Differences in clinical parameters between the two groups were evaluated by t-test and chi-square test. The Hardy–Weinberg Equilibrium (HWE) was checked (*p* < 0.05). Univariate logistic regression and Multivariable logistic regression were performed. Analysis of variance (ANOVA) test was also employed. Data are shown as mean with standard deviation (SD), *p* < 0.05 was considered as statistically significant.

The baseline clinical characteristics of the subjects with DR and without DR are shown in Table 1. The two groups were matched for age, sex, age at onset of T2DM, blood pressure and oral hypoglycaemics. Patients with DR had a higher HbA1c, fasting glucose, a more adverse family history, and were more likely to be needing insulin. Table 2 shows molecular genetics for the E23K SNP in *KCNJ11*. Variations in nucleotide make-up were equally distributed between the groups, and the genotype distributions of rs5219 alleles in the two groups were in Hardy–Weinberg equilibrium. Multivariate regression analysis with a DR/noDR as a dependent variable, and E23K genotype, age, sex, age of onset for diabetes, FBS, PBS, PBD, type of medicine, insulin injection, etc.) as independent variables. This indicated that HbAlc with adjusted OR of 1.84 (95% CI, 1.46–2.33, *p* < 0.01) and first-degree relatives of family history with an adjusted OR of 2.85 (95% CI, 1.45–5.58, *p* < 0.01) were significantly associated with DR, but the E23K is not an independent predictor of retinopathy.

**TABLE 1:** Clinical and demographic characteristics of the subjects.

	Without DR (control)	With DR (case)	*p*
Number of subjects	234	290	
Sex (female/male)	178/56	206/84	0.195
Age (years)	51.8 ± 9.3	52.7 ± 8.8	0.26
Fasting glucose (mmol/L)	8.2 ± 3.7	8.9 ± 3.9	0.04
HbAlc (%)	7.0 ± 1.4	8.4 ± 1.7	<0.01
Age of onset for diabetes (years)	41.5 ± 13.9	41.2 ± 18.5	0.3
SBP (mmHg)	124 ± 14	126 ± 14	0.21
DBP (mmHg)	75 ± 8	76 ± 9	0.64
Family History of T2DM			<0.01
First degree	143	217	
Second degree	16	24	
Third degree	71	44	
Requiring insulin	165 (70.5%)	172 (59.3%)	<0.01
Oral hypoglyaemics			
Metformin	188	230	0.567
Glibenclamide	23	24	
Metformin and Glibenclamide	23	36	

HbAlc, Hemoglobin A1c; SBP, systolic blood pressure; DBP, diastolic blood pressure; DR, diabetic retinopathy.

Data are presented as mean with SD or number with %.

**TABLE 2:** Allele and genotype distribution of the KCNJ11 E23K polymorphism in Diabetic Patients with DR and Patients without DR.

		Patients without DR (%)	Patients with DR (%)	OR (95%CI)	*p*
Allele (%)	G	295 (63%)	373 (64%)	1.05 (0.82–1.36)	0.66
	A	173 (37%)	207 (36%)		
Genotype (%)	GG	93 (39.7)	123 (42.4)	Ref.	Ref.
	GA	109 (46.6)	127 (43.8)	0.88 (0.60–1.27)	0.504
	AA	32 (13.7)	40 (13.8)	0.94 (0.55–1.61)	0.837
Hereditary model					
Co-dominant	(AA vs. GA)			1.05 (0.82–1.36)	0.66
Dominant	(AA‏+GA vs. GG)			0.89 (0.63–1.27)	0.537
Recessive	(AA vs. GA+‏GG)			1.00 (0.78–1.29)	0.969

Ref., reference; OR, odds ratio; CI 95%, confidence interval; DR, diabetic retinopathy.

Our data fail to support the hypothesis of a place for the E23K SNP in *KCNJ11*, in a Caucasian population, in contrast to the data from in a Chinese population (7). We deny the view that our finding is a false negative as we have sufficient power for this analysis. Several studies have explored the significant association between HbA1c, duration of diabetes and poor control of high blood pressure in the onset and progression of DR (8, 9). Unsurprisingly, in view of the pathophysiology of T2DM, there was a link between the HbA1c, fasting glucose, a first-degree family history of diabetes, and the need for insulin and DR.

Based on the reported association between *KCNJ11* polymorphisms and T2DM, we hypothesized that this gene could also be related to the risk of DR. Few studies have been performed on the association between polymorphism of *KCNJ11* and DR (10, 11). One of these showed that a strong association between the E23K and DR which has not been reported in previous studies (7), the current study is the second study to investigate the association of this polymorphism in the development of DR, that its results are not consistent with the first. This might be related to the separate gene pool of Chinese people compared to Iranians. According to our genotype-phenotype association study, T2DM subjects with retinopathy carrying GA genotypes, are at higher mean age to develop retinopathy than other genotypes. We point out that we investigated only one polymorphism of *KCNJ11*, so cannot rule out the involvement of this gene in the DR.

In summary, this represents an advance in biomedical sciences as it shows that the *KCNJ11* c.67A>G variant is not associated with diabetic retinopathy in a Caucasian population T2DM.

## Data Availability

The original contributions presented in the study are included in the article/supplementary material, further inquiries can be directed to the corresponding author.
